# Telencephalic morphogenesis is impaired in *Ftm/Rpgrip1l* KO mice

**DOI:** 10.1186/2046-2530-1-S1-P73

**Published:** 2012-11-16

**Authors:** C Laclef, M Catala, I Anselme, L Besse, S Schneider-Maunoury

**Affiliations:** 1 Laboratoire de Biologie du Développement, UMR7622 UPMC-CNRS, France

## 

Primary cilia have essential functions in vertebrate development and signaling. However, little is known about cilia function in brain morphogenesis, a process that is severely affected in human ciliopathies. Here, we study telencephalic morphogenesis in a mouse mutant for the ciliopathy gene *Ftm/Rpgrip1l* (also called *NPHP8*, *JBTS7* or *MKS5*). We have previously shown that E12.5 *Ftm-/-* telencephalic neuroepithelial cells lack primary cilia and that the telencephalon is ventralized at this stage. This dorso-ventral (DV) patterning defect leads to an ectopic localization of the olfactory bulb primordium, which consequently does not display morphological outgrowth at the end of gestation. This phenotype is reminiscent of Gli3 mutant and indeed correlates with a decreased production of the short, repressor form of Gli3 (Gli3R). We then demonstrate that the main function of primary cilia in the developing brain is to permit Gli3 prossessing, since introduction of Gli3R in *Ftm-/-* backgroung is sufficient to rescue DV patterning and OB morphological defects, despite the persistence of the cilia defects. In addition, we observed an impairement of the neocortex in Ftm mutant at the end of gestation. Cortical lamination is impaired, showing patches of very abnormal regions lying between regions that are less severely affected. This heterogeneity correlates with a disorganization of radial glial fibres and a reduction of the thickness of the ventricular layers and of the cortical plate. Investigating the developmental origin of these defects should shed light on aspects of physiopathology in human diseases.

